# The effect of script similarity on executive control in bilinguals

**DOI:** 10.3389/fpsyg.2014.01070

**Published:** 2014-09-29

**Authors:** Emily L. Coderre, Walter J. B. van Heuven

**Affiliations:** ^1^Department of Psychology, University of NottinghamNottingham, UK; ^2^Cognitive Neurology/Neuropsychology, Department of Neurology, Johns Hopkins University School of MedicineBaltimore, MD, USA

**Keywords:** bilingualism, executive control, script, Stroop task, Simon task

## Abstract

The need for executive control (EC) during bilingual language processing is thought to enhance these abilities, conferring a “bilingual advantage” on EC tasks. Recently, the reliability and robustness of the bilingual advantage has been questioned, with many variables reportedly affecting the size and presence of the bilingual advantage. This study investigates one further variable that may affect bilingual EC abilities: the similarity of a bilingual's two languages. We hypothesize that bilinguals whose two languages have a larger degree of orthographic overlap will require greater EC to manage their languages compared to bilinguals who use two languages with less overlap. We tested three groups of bilinguals with language pairs ranging from high- to low-similarity (German-English (GE), Polish-English (PE), and Arabic-English (AE), respectively) and a group of English monolinguals on a Stroop and Simon task. Two components of the bilingual advantage were investigated: an interference advantage, such that bilinguals have smaller interference effects than monolinguals; and a global RT advantage, such that bilinguals are faster overall than monolinguals. Between bilingual groups, these effects were expected to be modulated by script similarity. AE bilinguals showed the smallest Stroop interference effects, but the longest overall RTs in both tasks. These seemingly contradictory results are explained by the presence of cross-linguistic influences in the Stroop task. We conclude that similar-script bilinguals demonstrated more effective domain-general EC than different-script bilinguals, since high orthographic overlap creates more cross-linguistic activation and increases the daily demands on cognitive control. The role of individual variation is also discussed. These results suggest that script similarity is an important variable to consider in investigations of bilingual executive control abilities.

## Introduction

Human language is a remarkably complicated ability, requiring timely coordination and recruitment of cognitive resources. Understandably, acquiring and using a second language is considerably more difficult, especially in adulthood. One question that has arisen out of research into the cognitive effects of bilingualism is how bilinguals control their two languages. After decades of research, it is now generally accepted that even in completely monolingual language processing contexts, both of a bilingual's languages are activated in parallel. This *non-selective* access to the bilingual lexicon is supported by a wealth of evidence demonstrating that the second language (L2) can have both detrimental and facilitatory effects on first language (L1) processing, and vice versa (e.g., Soares and Grosjean, 1984; Poulisse and Bongaerts, 1994; van Heuven et al., 1998, 2008; Colomé, 2001; van Hell and Dijkstra, 2002; Thierry and Wu, 2004, 2007; Rodriguez-Fornells et al., 2005; Kerkhofs et al., 2006; Midgley et al., 2008; Martin et al., 2009; Degani and Tokowicz, 2010; see reviews in Dijkstra and van Heuven, 2002; Kroll et al., 2006, 2012; Brysbaert and Duyck, 2010).

As a result, bilinguals must constantly exert control over their languages to manage these cross-linguistic effects resulting from non-selective lexical access. *Executive control* is an umbrella term that refers to processes such as managing distracting information, overcoming a habitual response, or switching between tasks or rules. Neuroimaging research has demonstrated that bilinguals activate brain areas involved in executive control when processing one or both of their languages (e.g., Rodriguez-Fornells et al., 2005; Hernandez and Meschyan, 2006; van Heuven et al., 2008; Parker Jones et al., 2011), suggesting an interdependence of executive control and language processing in the bilingual brain.

Although it has been shown that executive control is involved in language processing even in monolingual speakers (e.g., Ye and Zhou, 2009), bilinguals must additionally manage the cross-linguistic influences arising from non-selective lexical access (Costa and Sebastián-Gallés, 2014). This additional recruitment of executive control on a daily basis is thought to confer more efficient cognitive processing abilities for bilinguals compared to monolinguals (see Green and Abutalebi, 2013 for an extended discussion), a phenomenon known as the *bilingual advantage*. There is now extensive empirical evidence demonstrating superior performance, across a range of executive control domains, for bilinguals compared to monolinguals (see Bialystok, 2009, 2011; Bialystok et al., 2009; Hilchey and Klein, 2011; Tao et al., 2011; Kroll and Bialystok, 2013 for reviews). Here, we refer to the hypothesis that the interdependence of executive control and language processing results in enhanced cognitive abilities in bilinguals as the *bilingual cognitive advantage hypothesis*.

Recently, however, the reliability of the bilingual advantage has been questioned (e.g., Hilchey and Klein, 2011; Paap and Greenberg, 2013; Duñabeitia et al., 2014). In a recent review, Hilchey and Klein (2011) proposed that a “global reaction time” advantage in conflict tasks (i.e., faster reaction times (RTs) for bilinguals than monolinguals on all trials, both incongruent and congruent) is a more common finding than a bilingual “interference advantage” (i.e., smaller conflict effects when comparing incongruent and congruent trials; e.g., Bialystok et al., 2004, 2005a; Martin-Rhee and Bialystok, 2008; Costa et al., 2009; although see Bialystok et al., 2008). Hilchey and Klein (2011) proposed two hypotheses reflecting subdivisions of the bilingual advantage in executive processing: the “bilingual inhibitory control advantage,” or *BICA hypothesis*; and the “bilingual executive processing advantage,” or *BEPA hypothesis*.

The BICA hypothesis is based on a theory that bilinguals recruit inhibitory control to manage cross-linguistic interference during language production (Green, 1998). As a result, it is hypothesized that bilinguals have more efficient inhibitory processes in the presence of conflict (i.e., incongruent trials) and thus exhibit smaller interference effects (incongruent vs. control conditions). The finding of smaller interference effects for bilinguals than for monolinguals is referred to as the *bilingual interference advantage*. In contrast, the BEPA hypothesis proposes that bilingualism confers a domain-general advantage in executive processing which is not restricted to the presence of conflict (Martin-Rhee and Bialystok, 2008; Costa et al., 2009). For example, bilinguals may be more efficient at monitoring the environment for conflict (Costa et al., 2009) or at top-down guidance of attention (Hernández et al., 2012). Such a domain-general enhancement of executive processing predicts faster processing on all trial types, leading to a *global RT advantage* such that bilinguals have faster RTs on all conditions, congruent as well as incongruent, compared to monolinguals. Importantly, because the BICA hypothesis proposes that the bilingual interference advantage arises from inhibitory control in the presence of conflict, it predicts that in the absence of conflict there should be no difference between groups. It thus cannot account for findings of a global RT advantage. Both the interference advantage and global RT advantage will be investigated in the current study; references to a more general “bilingual advantage” are meant to incorporate both of these subdivisions.

The wealth of research on the bilingual advantage in recent years has demonstrated that many individual variables can affect the magnitude and presence of the bilingual advantage effect, including age (Craik and Bialystok, 2006), vocabulary knowledge (Bialystok and Feng, 2009), social-economic status (Morton and Harper, 2007; Carlson and Meltzoff, 2008), L2 proficiency level (Bialystok et al., 2006a), and frequency of language switching (Festman et al., 2010; Soveri et al., 2011). The current study investigates another variable that may affect bilingual executive control abilities: the similarity of a bilingual's two languages.

Research with monolinguals has demonstrated that specific characteristics of orthography and phonology can influence how a language is processed at various cognitive and linguistic levels. For example, debates remain concerning the role that phonology plays in visual word recognition, and how this influence might differ across writing systems (e.g., Saalbach and Stern, 2004; Perfetti et al., 2005). Neural and electrophysiological data demonstrates that language processing differs between shallow orthographies (such as Italian and Finnish, with regular grapheme-phoneme conversion rules) and deep orthographies (such as English, French and Arabic, with many irregular grapheme-phoneme mappings; e.g., Meschyan and Hernandez, 2006; Bar-Kochva, 2011). For example, Bar-Kochva (2011) observed larger N170 effects for shallow orthographies than for deep orthographies. The neural organization of language is also shaped by writing system (e.g., Sakurai et al., 2000; Tan et al., 2001, 2005b; Bolger et al., 2005; Perfetti et al., 2007; Coderre et al., 2008; Nelson et al., 2009; Bick et al., 2011). For example, Chinese, being a more spatial logographic writing system, recruits a bilateral network for language processing, whereas language networks for alphabetic writing systems are left-lateralized (see Bolger et al., 2005 and Tan et al., 2005a for meta-analyses). Differences have also been observed at the level of production: picture naming latencies are influenced by a language's specific lexical and grammatical characteristics (Bates et al., 2003).

The cognitive effects of these linguistic differences become more complicated in the case of bilingualism: the two languages that a bilingual uses may differ drastically in their language-specific characteristics. Most work on cross-linguistic influences has been conducted with language pairs from the same writing system (e.g., Dutch and English). Despite the above-mentioned variations in linguistic processing characteristics, different-script languages also experience cross-linguistic activation (Sumiya and Healy, 2004; Hoshino and Kroll, 2008; Zhang et al., 2011). For example, in a picture naming task with Japanese-English bilinguals Hoshino and Kroll (2008) reported cross-linguistic effects of phonology, suggesting that even though the non-target language had a completely different writing system, it was activated in parallel and influenced processing in the target language.

Importantly, however, orthographic similarity may modulate the amount of cross-linguistic activation. The Bilingual Interactive Activation + (BIA+) model is one of the foremost models of bilingual word recognition. This model simulates visual word recognition in alphabetic writing systems by coding letter positions within words. The BIA+ model proposes that the degree of orthographic overlap between two languages determines the amount of cross-linguistic bottom-up activation (Dijkstra and van Heuven, 2002):

“The larger the overlap between the input string and a representation in the mental lexicon, the more the internal representation is activated… if the two languages differ with respect to their input codes (e.g., letter sets), the activated set of [orthographic] neighbors may become much smaller” (Dijkstra and van Heuven, 2002, pp. 182–183).

Therefore, according to this model, two alphabetic languages with higher amounts of orthographic overlap (i.e., more overlapping letters) would lead to greater cross-linguistic influences. Furthermore, cross-linguistic influences might be exaggerated by the fact that two same-script languages may also share a high number of orthographic neighbors (words that differ by one letter) and homographs (words that have the same spelling across languages). The BIA+ model would therefore predict that bilinguals whose two languages have a high degree of overlap (referred to here as *same-script bilinguals*), such as German and English, manage greater amounts of cross-linguistic activation than bilinguals whose languages have less overlap (referred to here as *different-script bilinguals*), such as Chinese and English. As a result, bilingual executive control abilities, and the magnitude of the bilingual cognitive advantage, may be modulated by script similarity.

Significant bilingual advantages have been found for both same-script bilinguals (e.g., Spanish-Catalan: Costa et al., 2008, 2009; Garbin et al., 2010; French-English: Poulin-Dubois et al., 2011) and different-script bilinguals (e.g., Chinese-English: Bialystok, 1999; Kuo and Anderson, 2012). However, very few studies have systematically investigated the effects of script similarity, and research on the bilingual advantage rarely takes this variable into consideration. A surprising amount of previous work even includes bilinguals from a diverse assortment of native languages (Bialystok and Shapero, 2005; Bialystok et al., 2006a,b, 2008; Bialystok and Feng, 2009; Bialystok, 2010; Luk et al., 2010; Bartolotti et al., 2011). For example, the bilingual population in Bialystok et al. (2008) included native speakers of 24 different languages, including Spanish, German, Greek, Korean, Hebrew, Arabic, Tamil, Latvian, and Cantonese.

Among studies testing such heterogeneous bilingual populations, there is evidence both for (Bialystok and Shapero, 2005; Bialystok et al., 2008) and against (Bialystok et al., 2006b; Luk et al., 2010) the presence of a bilingual advantage. Similarly, among studies testing homogeneous populations there is also evidence both for (Bialystok, 1999; Bialystok et al., 2004; Carlson and Meltzoff, 2008; Costa et al., 2008, 2009; Garbin et al., 2010; Blumenfeld and Marian, 2011; Poulin-Dubois et al., 2011; Kuo and Anderson, 2012) and against (Morton and Harper, 2007; Kousaie and Phillips, 2012) a bilingual advantage. However, very few studies have systematically manipulated script similarity to evaluate whether and how this factor affects the bilingual advantage.

The current study investigated how script similarity modulates bilingual executive control abilities by testing three bilingual groups with differing amounts of overlap between their two languages: German L1 and English L2 (high amounts of both orthographic and phonological overlap); Polish L1 and English L2 (both alphabetic writing systems but with less orthographic and phonological overlap); and Arabic L1 and English L2 (both alphabetic writing systems but no orthographic and very little phonological overlap). In addition, a group of English monolinguals was included to evaluate the presence of a bilingual advantage. The participants were tested on a Stroop task (in both the L1 and L2 for bilinguals; in English only for monolinguals) as well a non-linguistic Simon task. The bilingual advantage was assessed in light of both the BICA and BEPA hypotheses. When comparing all groups of bilinguals to the monolinguals, the BICA hypothesis would predict smaller interference effects for bilinguals, whereas the BEPA hypothesis would predict faster global RTs for bilinguals.

In addition, we predicted that bilingual executive control abilities would be modulated by script similarity. Two hypotheses were proposed. *Hypothesis 1*, based on the BIA+ model, predicted that a large amount of cross-linguistic activation occurs for similar language pairs like English and German due to the high degree of phonological and orthographic overlap. This would require more cognitive control on a daily basis for same-script bilinguals, which would enhance control abilities such that similar-script bilinguals should show more effective executive control abilities compared to different-script bilinguals. More specifically, the BICA hypothesis would predict that same-script bilinguals have enhanced inhibitory control abilities and should therefore show larger interference advantages (i.e., smaller interference effects). The BEPA hypothesis would propose that the greater amount of cross-linguistic activation enhances processes of monitoring for and selecting the target language, leading to enhanced executive processing abilities for same-script bilinguals, and manifesting as a larger global RT advantage (i.e., faster global RTs) compared to different-script bilinguals. Therefore, Hypothesis 1 predicted a *positive* relationship between script similarity and executive control abilities, with more effective executive control (smaller interference/global RT effects) for German-English bilinguals, followed by Polish-English and Arabic-English bilinguals, respectively.

Hypothesis 2 was based on the scant existing literature on script effects in the bilingual advantage. Two studies have explicitly considered the influences of script differences in bilingual cognitive control (Bialystok et al., 2005a; Linck et al., 2008). Linck et al. (2008) hypothesized that different-script bilinguals are at an advantage because they can use script as a cue to help restrict lexical selection (Guo et al., 2005; Hoshino and Kroll, 2008). This strategy is not as useful in same-script bilinguals, who must rely more on executive control for managing linguistic competition. Testing Japanese-English and Spanish-English bilinguals on a Simon task, Linck et al. (2008) therefore predicted more effective cognitive control, and a larger bilingual advantage, for same-script bilinguals (as in Hypothesis 1 in the current study). However, they observed no group differences in Simon effects; in fact, when looking at the data from bilinguals who were tested in an L2 context, greater inhibitory control abilities were found for different-script bilinguals, in contrast to their predictions. Linck et al. speculated that this effect may be due to group differences in code-switching frequency: because Japanese-English bilinguals generally code-switch less often, this group may have better-developed language control mechanisms, whereas Spanish-English bilinguals, who generally code-switch more often, may have less experience with language control. Also using a Simon task, Bialystok et al. (2005a) tested two bilingual groups: French-English and Cantonese-English (although they did not provide a rationale for why script similarity was manipulated, nor why they chose those specific language pairs). Behaviorally, the French-English bilinguals did not differ from monolinguals, but Cantonese-English bilinguals exhibited a global RT advantage compared to monolinguals. This suggests that different-script bilinguals had more effective executive control than same-script bilinguals. However, the authors did not interpret this group difference beyond ascribing it to sampling variability.

In sum, these two studies both suggest that bilingual executive control abilities may decrease with increasing script similarity. Therefore, in contrast to Hypothesis 1, *Hypothesis 2* proposed a *negative* relationship between script similarity and bilingual cognitive control abilities, predicting a larger bilingual advantage (i.e., smaller interference/global RT effects) for Arabic-English bilinguals, followed by Polish-English and German-English bilinguals, respectively. Note that for both Hypothesis 1 and Hypothesis 2, the performance of the Polish-English bilingual group was expected to fall between German-English and Arabic-English bilinguals. Furthermore, in line with previous literature showing bilingual advantages on Stroop and Simon tasks, monolinguals were expected to show the worst executive control in both hypotheses. Therefore, we predicted a bilingual advantage in both interference effects and overall RTs, as well as a modulation of these effects between bilingual groups according to script differences.

These hypothesis-specific patterns were expected for Simon interference effects and for the global RT effects in both the Stroop and Simon tasks. However, because the Stroop task explicitly involves language, Stroop interference effects may follow a different pattern. Previous work with the bilingual Stroop task (in which the written word is in the L1 and bilinguals must respond vocally in the L2, or vice versa) has demonstrated larger Stroop interference effects with increasing language similarity (e.g., Brauer, 1998; van Heuven et al., 2011). This would predict the greatest Stroop interference effects for German-English bilinguals, in contrast to Hypothesis 1 but as predicted by Hypothesis 2. To assess executive control abilities independently of cross-linguistic interference effects, we also examine conflict effects in the non-linguistic Simon task as a measure of the bilingual interference advantage. To assess the bilingual global RT advantage, we compared not just the global RTs (collapsed over congruent, incongruent, and control conditions) but also RTs to the control condition (a symbol string in the Stroop task and a centrally-presented square in the Simon task) between groups to eliminate any linguistic influences.

### Materials and methods

#### Participants

Participant demographic information is presented in Table 1. All participants were right-handed and reported no color-blindness. Three groups of bilinguals were included: German-English (GE; *n* = 19), Polish-English (PE; *n* = 22), and Arabic-English (AE; *n* = 17). All bilinguals lived in England at the time of testing and all considered English to be their second language. Participants completed a language background questionnaire prior to testing and two vocabulary assessments (X-Lex and Y-Lex; see Section Procedure). The bilingual groups did not differ statistically on their self-rated proficiency (all *p*'s > 0.21), years of English experience (all *p*'s > 0.47), or English age of acquisition (all *p*'s > 0.17), although Arabic bilinguals had significantly lower scores on the English vocabulary measures compared to the GE and PE groups (all *p*'s < 0.05). The monolingual participants were 18 native English speakers (Table 1). Some reported learning other languages (*n* = 9), but none considered themselves fluent in any other language besides English.

**Table 1:** **Demographic and proficiency information for all participants (F, female, M, male)**.

	**German-English (GE)**	**Polish-English (PE)**	**Arabic-English (AE)**	**Monolingual**
*n*	19	22	17	18
Age	26 (6)	25 (5)	26 (4)	21 (2)
Gender	11 F, 8 M	13 F, 9 M	9 F, 8 M	9 F, 9 M
X-Lex score				
Raw	4875 (170)	4891 (128)	4671 (289)	4976 (31)
Adjusted	4514 (402)	4436 (369)	3626 (833)	4550 (511)
Y-Lex score				
Raw	3672 (961)	3684 (561)	2926 (1054)	4406 (396)
Adjusted	3353 (934)	2905 (808)	2000 (868)	3706 (912)
Age of first L2 contact	9.6 (2.3)	8.9 (3.1)	7.9 (4.5)	–
Years experience	14.4 (5.8)	13.4 (5.0)	12.9 (5.9)	–
Self-rated L2 proficiency				
Speaking	8.7 (1.1)	8.7 (1.2)	8.2 (1.3)	–
Listening	8.9 (1.2)	8.8 (1.3)	8.7 (1.0)	–
Reading	8.9 (1.2)	9.1 (1.1)	8.6 (0.9)	–
Writing	8.3 (1.4)	8.5 (1.1)	8.1 (1.3)	–
Overall	8.7 (1.1)	8.8 (1.0)	8.4 (1.0)	–

*X-Lex and Y-Lex scores range from 0 to 5000 in 100-point increments. The adjusted score accounts for false alarms*.

#### Materials and design

##### The Stroop task

Word stimuli for the English Stroop task consisted of the words “red,” “green,” and “blue” in lowercase letters. Corresponding word stimuli for the L1 task were: German words “rot,” “grün,” “blau”; Polish words “czerwony,” “zielony,” “niebieski”; and Arabic words 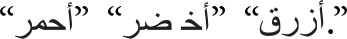 The non-linguistic control condition was a symbol string (“%%%%”). All stimuli were printed in white ink on a black background. Color stimuli consisted of red, green and blue filled rectangles (284 × 142 pixels) with a smaller black-filled rectangle centered inside (142 × 42 pixels). Congruent stimuli presented the same word and color (e.g., “red” surrounded by a red rectangle), whereas incongruent stimuli presented non-matching words and colors (e.g., “green” surrounded by a blue rectangle). Control stimuli presented “%%%%” surrounded by red, green or blue rectangles. Participants were asked to ignore the word and indicate the color of the rectangle by pressing a corresponding keyboard button (right index finger for red, right middle finger for green, right ring finger for blue). Participants were given a practice session before testing to familiarize themselves with the color-to-button mappings.

##### The Simon task

Stimuli in the Simon task consisted of blue and red squares (60 × 60 pixels) on a white background, presented either in the center or slightly to the left (42% of horizontal) or right (58% of horizontal) of center. Participants responded to the color of the square with a keyboard button response (left index finger for blue, right index finger for red). Congruent conditions presented the colored square to the same side as the resulting response (e.g., a blue square, requiring a left-hand response, on the left side of the screen). Incongruent conditions presented incompatible lateralization and response pairs (e.g., a blue square, requiring a left-hand response, on the right side of the screen). The control condition presented the colored square in the center of the screen, thus did not contain any conflicting information.

#### Procedure

Ethics approval was granted by the Research Ethics Committee in the School of Psychology at the University of Nottingham. Informed consent was obtained from all participants prior to experimental testing. Before testing, participants completed an online language background questionnaire, a short color-blind test, and two vocabulary tests estimating high-frequency (1K-5K: X-Lex: Meara, 2005) and low-frequency (5K-10K: Y-Lex: Meara and Miralpeix, 2006) word knowledge. Monolingual participants performed one session, consisting of the English Stroop task and the Simon task. Bilingual participants performed two experimental sessions on consecutive days; each session consisted of the Simon task and the Stroop task in one language (L1 or L2). The behavioral data was collected during an EEG session (data not reported here; Coderre, 2012). The order of task and of Stroop language administration was counterbalanced across participants. In the second session, bilinguals performed a picture-naming task in both of their languages (data not reported here).

Stimuli were presented using E-Prime. In the Stroop task, a stimulus onset asynchrony (SOA) of −200 ms was used, such that the word appeared on the screen alone for 200 ms and then was surrounded by the colored rectangle. In previous work (Coderre et al., 2011, 2013; Coderre and van Heuven, 2013) we have observed that this SOA generates the largest interference effects when using a manual response modality. Therefore, this SOA might be expected to generate the largest differences between groups, should they exist. Two other SOAs of −400 ms and 0 ms were also tested during the course of this experiment, but for clarity these data are not reported here (Coderre, 2012). SOA was blocked and counterbalanced between participants. Participants performed four blocks (approximately 4 min each) of the Stroop task with the −200 ms SOA. Each block consisted of 54 trials, of which 18 were congruent, 18 control and 18 incongruent, resulting in 216 trials total. In each trial, a fixation cross appeared for 500 ms, followed by a blank screen for 300 ms. The word then appeared on the screen for 200 ms and was then surrounded by the colored rectangle. Once both stimuli were presented they remained on the screen for 1000 ms. Participants were instructed to respond to the color of the rectangle as quickly and accurately as possible. A blank screen was presented following each trial at an interstimulus interval (ISI) varying from 1500 to 2000 ms. Congruency was randomized within blocks.

The Simon task was also presented in E-Prime. A practice session consisting of 24 stimuli was first administered, followed by the experimental blocks. Each experimental block was approximately 2 min long. Bilinguals performed 3 blocks in each session, for 6 blocks total. Monolinguals performed 6 blocks during their single session. Each block consisted of 42 trials (14 each of congruent, control, and incongruent), creating 252 total trials for each participant. Congruency was randomized within block. In each trial, a fixation cross was presented for 350 ms, followed by a blank screen for 150 ms, then the colored square for 750 ms. A blank screen was then presented with an inter-trial interval of 850 ms.

#### Data analysis

Linear mixed effects modeling was performed with the *lme4* package (version 1.1-7, Bates et al., 2014) in R version 3.1.1 (R Core Team, 2014). To address non-normality of the RT distributions, RTs were transformed using a reciprocal transformation (−1000/RT; see Kliegl et al., 2010). The initial models included fixed effects of congruency (control, congruent, incongruent); group (monolinguals, AE, GE, PE); and trial number (centered; to account for the possibility of fatigue or learning effects over the course of the experiment); as well as interactions between these predictors. We started with a maximal random effects model (Barr et al., 2013) that included varying intercepts for subjects and items, and random slopes for the effects of: group by item, congruency by item, trial number by item, congruency by subject, and trial number by subject. (Random slopes for group by subject were not considered because group is a between-subject variable.) When the maximal model failed to converge, the random effects structure was simplified using a backward model selection procedure. We removed first the random slopes for each predictor (but kept the random intercepts of subject and item), then the interactions between the fixed effects, then the main effects. At each step, the simplified model was compared to the preceding model using a Chi-squared test. If the test was not significant, we proceeded with the simplified model. All models were estimated using maximum likelihood.

Simple contrast coding was used for all comparisons. Fixed effects estimates for the comparisons of interest (i.e., all possible differences between groups) were performed using function *glht* in the *multcomp* package (Hothorn et al., 2008), which provides multiple simultaneous comparisons based on a normal approximation. *t*- or *z*-scores greater than 2 were interpreted as significant effects corresponding to an alpha of 0.05 or lower (Meier and Kane, 2013; see also Gelman et al., 2012). For significant between-group effects, we also calculated Cohen's *d* values based on the means and standard deviations for each group.

## Results

Incorrect responses and outliers (RTs less than 250 ms or greater than 2000 ms; presented in Table 2) were removed before starting the linear mixed effects modeling. After fitting the mixed effects models, visual inspection of the fitted vs. residual values was performed. If the distribution looked heteroskedastic, additional outliers of ±2.5 *SD*s (of the entire dataset) were removed and the model was refit. The percentages of errors and outliers are reported in Table 2. Because error rates were fairly low, they are not analyzed here.

**Table 2:** **Percentages of errors, range of errors across conditions, and outliers (after fitting the linear mixed effects models) for each group and language**.

	**% Errors**	**% Errors per condition (range)**	**% Outliers**
**GERMAN-ENGLISH**			
Stroop			
L1	4.6	0.3–0.8	2
L2	3.7	0.3–0.5	2
Simon	6.8	2.0–2.9	2.6
**POLISH-ENGLISH**			
Stroop			
L1	4.6	0.4–0.7	2.1
L2	5.2	0.5–0.8	2
Simon	6.2	1.4–3.1	2.9
**ARABIC-ENGLISH**			
Stroop			
L1	6.3	0.6–0.9	1
L2	7.3	0.7–0.9	1
Simon	7.2	2.0–3.2	1.6
**MONOLINGUALS**			
Stroop	3.7	0.3–0.5	2.1
Simon	7.1	1.6–3.6	1.7

### Stroop task

#### Bilingual L1 vs. English monolinguals

**Figure 1:**
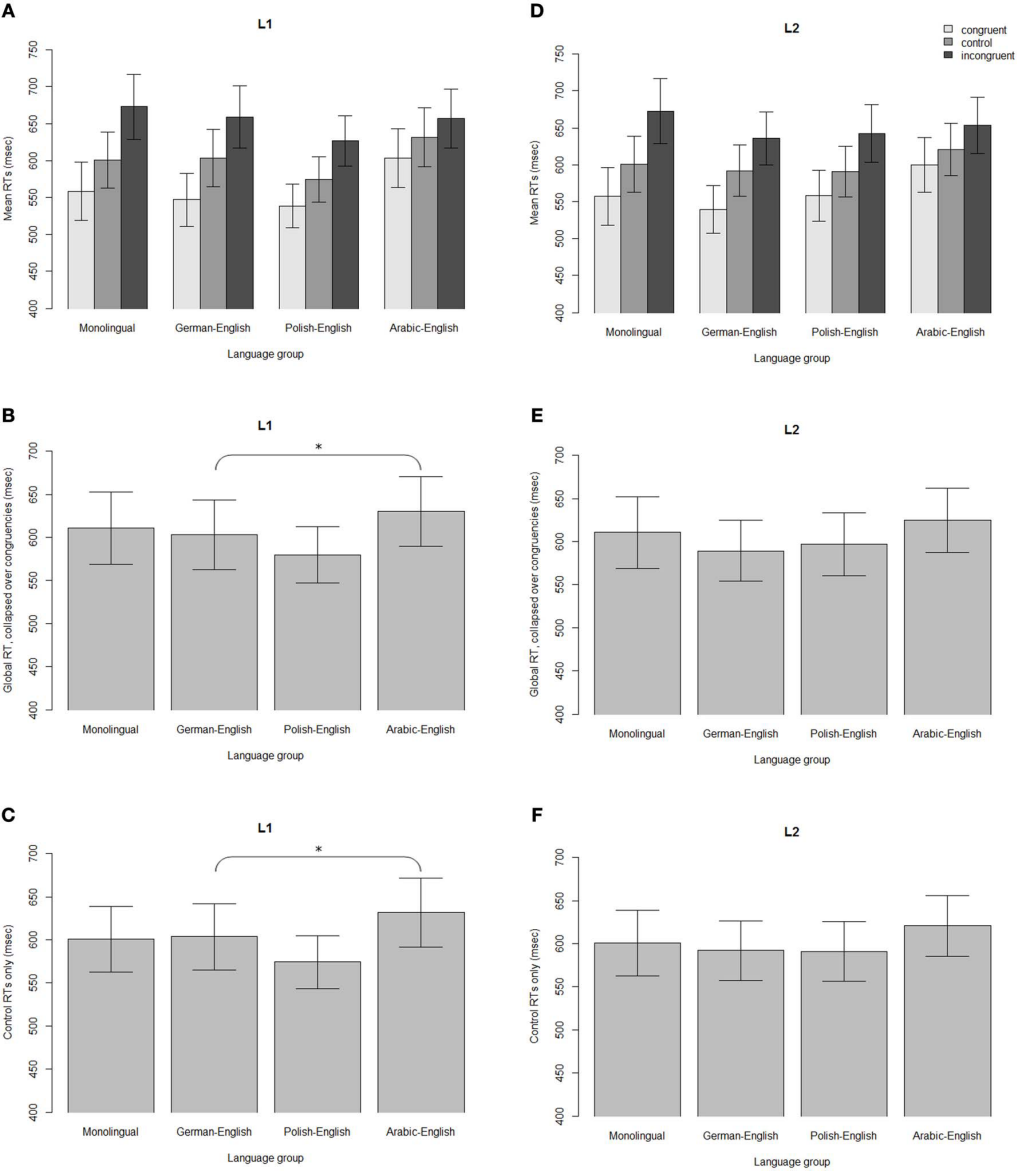
**Top panels: Mean RTs (ms) in the Stroop task for each group in the L1 (A) and L2 (D)**. Middle panels: Mean RTs, collapsed over all congruencies, for the L1 **(B)** and L2 **(E)**. Bottom panels: Mean RT for the control condition only, for the L1 **(C)** and L2 **(F)**. Significant differences between groups (*z* > 2) are indicated with an asterisk.

The mean RTs for each group and congruency are presented in Figure 1A. The final model for the RTs in the L1 Stroop task is presented in Table 3. As can be seen in Table 3, there was a significant interference effect (collapsed over groups) such that RTs were slower for incongruent trials (*M* = 653 ms, *SE* = 20 ms) than control trials (*M* = 600 ms, *SE* = 18 ms; *t* = 4.62). A significant facilitation effect was also found, such that RTs were faster for congruent trials (*M* = 559 ms, *SE* = 18 ms) than control trials (*t* = 4.37).

**Table 3:** **Results of the mixed-effects analysis of the L1 Stroop RTs for bilinguals and monolinguals**.

**Random effects**		**Variance**	***SD***
Subject	Intercept	0.047596	0.21817
	congruent	0.006124	0.07826
	incongruent	0.002445	0.04944
Item	Intercept	0.002094	0.04576
	German	0.002972	0.05451
	Polish	0.002789	0.05281
	Arabic	0.001339	0.03659
Residual		0.109710	0.33123

**Table:** 

**Fixed effects**	**Estimate**	***SE***	***t*-value**
Intercept	−1.760e+00	2.764e-02	−63.66*
TrialNr	−2.213e-04	4.352e-05	−5.08*
Congruency: congruent	−1.410e-01	3.226e-02	−4.37*
Congruency: incongruent	1.374e-01	2.972e-02	4.62*
Group: GE	−1.762e-02	7.575e-02	−0.23
Group: PE	−6.752e-02	7.333e-02	−0.92
Group: AE	8.815e-02	7.880e-02	1.12
TrialNr * congruent	−6.797e-05	1.061e-04	−0.64
TrialNr * incongruent	8.838e-05	1.067e-04	0.83
TrialNr * GE	6.153e-04	1.230e-04	5.00*
TrialNr * PE	6.760e-04	1.186e-04	5.70*
TrialNr * AE	7.566e-04	1.288e-04	5.87*
Congruent * GE	−2.834e-02	6.725e-02	−0.42
Incongruent * GE	−4.036e-02	5.352e-02	−0.75
Congruent * PE	4.069e-02	6.593e-02	0.62
Incongruent * PE	−3.099e-02	5.232e-02	−0.59
Congruent * AE	7.947e-02	6.645e-02	1.20
Incongruent * AE	−1.123e-01	5.051e-02	−2.22 *
TrialNr * congruent * GE	7.720e-04	3.007e-04	2.57*
TrialNr * incongruent * GE	6.639e-05	3.016e-04	0.22
TrialNr * congruent * PE	2.057e-04	2.901e-04	0.71
TrialNr * incongruent * PE	−1.235e-04	2.896e-04	−0.43
TrialNr * congruent * AE	5.754e-04	3.134e-04	1.84
TrialNr * incongruent * AE	−5.454e-04	3.163e-04	−1.72

*Note that the reference levels for this model are the control condition (for congruency) and the monolingual group (for group). Thus, the fixed effect of “congruency: congruent” compares control vs. congruent (i.e., facilitation), the fixed effect of “group: AE” compares monolinguals vs. AE, etc. Significant fixed effects (t > 2) are marked with an asterisk*.

**Figure 2:**
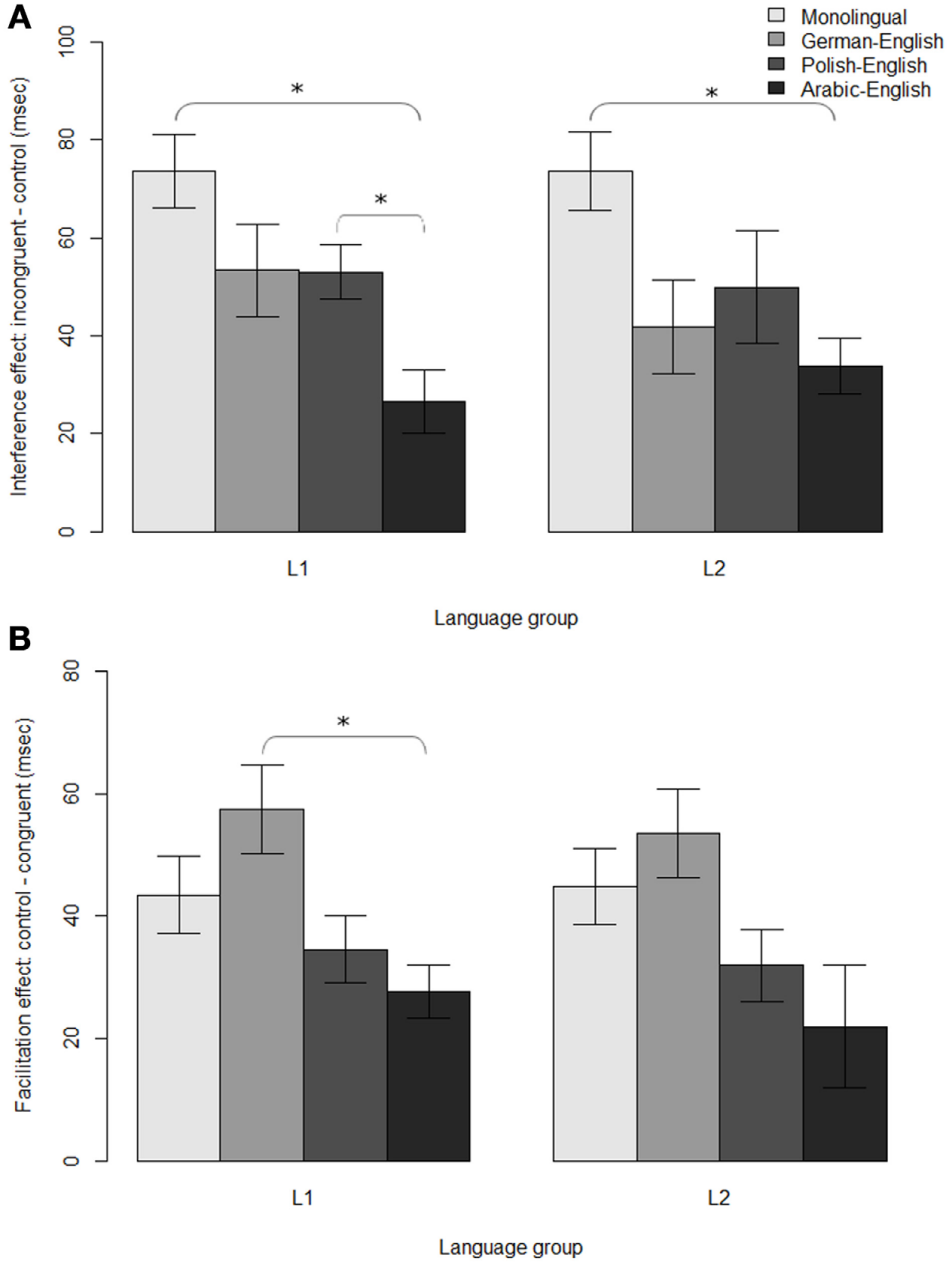
**(A)** Interference effects and **(B)** facilitation effects for each group and language in the Stroop task (the same monolingual data was compared against each language). Significant differences between groups (*z* > 2) are indicated with an asterisk.

Group comparisons (Table 4) showed significantly smaller interference effects for AE bilinguals (*M* = 27 ms, *SE* = 6 ms) compared to both monolinguals (*M* = 74 ms, *SE* = 8 ms; *z* = 2.22; *d* = 1.58; Figure 2A) and to PE bilinguals (*M* = 53 ms, *SE* = 5 ms; *z* = 2.35; *d* = 1.08). Facilitation effects were also significantly smaller for AE bilinguals (*M* = 28 ms, *SE* = 4 ms) than for GE bilinguals (*M* = 57 ms, *SE* = 7 ms; *z* = 2.28; *d* = 1.18; Figure 2B).

**Table 4:** **Fixed effects of group comparisons for L1 Stroop RTs (based on model presented in Table 3)**.

	**Estimate**	***SE***	***z*-value**
**GLOBAL RTs**			
Monolingual vs. German	−1.762e-02	7.575e-02	−0.233
Monolingual vs. Polish	−6.752e-02	7.333e-02	−0.921
Monolingual vs. Arabic	8.815e-02	7.880e-02	1.119
German vs. Polish	4.990e-02	6.946e-02	0.718
German vs. Arabic	1.058e-01	7.568e-02	1.398
Polish vs. Arabic	1.557e-01	7.325e-02	2.125*
**INTERFERENCE EFFECTS**			
Monolingual vs. German	−4.036e-02	5.352e-02	−0.754
Monolingual vs. Polish	−3.099e-02	5.232e-02	−0.592
Monolingual vs. Arabic	−1.123e-01	5.051e-02	−2.223*
German vs. Polish	−9.374e-03	2.978e-02	−0.315
German vs. Arabic	−7.191e-02	3.647e-02	−1.971
Polish vs. Arabic	−8.128e-02	3.453e-02	−2.354*
**FACILITATION EFFECTS**			
Monolingual vs. German	−2.834e-02	6.725e-02	−0.421
Monolingual vs. Polish	4.069e-02	6.593e-02	0.617
Monolingual vs. Arabic	7.947e-02	6.645e-02	1.196
German vs. Polish	−6.903e-02	3.932e-02	−1.755
German vs. Arabic	1.078e-01	4.738e-02	2.276*
Polish vs. Arabic	3.878e-02	4.536e-02	0.855

*Note that the monolingual comparisons are identical to those presented in Table 3. Significant effects (z > 2) are marked with an asterisk*.

There was also a difference in overall RTs such that, when collapsing over all congruencies, AE bilinguals were significantly slower (*M* = 630 ms, *SE* = 40 ms) than PE bilinguals (*M* = 580 ms, *SE* = 32 ms; *z* = 2.13; *d* = 0.32; Figure 1B). To further examine non-conflict-specific effects of executive processing between groups, we also compared RTs of the control conditions between groups for the Stroop task in the L1 (Figure 1C). For this analysis, a separate mixed effects model was fit using only trial number and group, and their interaction, as fixed effects. Random effects included random intercepts for subject and item and random slopes for the effect of group by item, as well as interactions between trial number, congruency, and group. The results showed significantly slower control RTs for AE bilinguals (*M* = 632 ms, *SE* = 40 ms) than for PE bilinguals (*M* = 574 ms, *SE* = 31 ms; *z* = 2.34; *d* = 0.37; Figure 1C).

#### Bilingual L2 (English) vs. English monolinguals

The mean RTs for each group and congruency are presented in Figure 1D. The final model for the RTs in the bilingual L2 (English) and English monolinguals is presented in Table 5. The results revealed a significant interference effect when collapsed over groups, such that RTs were significantly slower for incongruent trials (*M* = 650 ms, *SE* = 20 ms) than control trials (*M* = 600 ms, *SE* = 18 ms; *t* = 4.48; Table 5, Figure 1D). A significant facilitation effect was also found, with significantly faster RTs for congruent trials (*M* = 562 ms, *SE* = 18 ms) than control trials (*t* = 4.02).

Table 5**Results of the mixed-effects analysis of the L2 Stroop RTs for bilinguals and monolinguals**.

**Table:** 

**Random effects**		**Variance**	***SD***
Subject	Intercept	0.049214	0.22184
	congruent	0.008432	0.09183
	incongruent	0.004877	0.06983
Item	Intercept	0.001495	0.03866
	German	0.003430	0.05857
	Polish	0.004794	0.06924
	Arabic	0.002739	0.05234
Residual		0.103984	0.32246

**Table:** 

**Fixed effects**	**Estimate**	***SE***	***t*-value**
Intercept	−1.758e+00	2.826e-02	−62.21*
TrialNr	−2.873e-04	4.216e-05	−6.81*
Congruency: congruent	−1.362e-01	3.391e-02	−4.02*
Congruency: incongruent	1.308e-01	2.921e-02	4.48*
Group: GE	−3.844e-02	7.548e-02	−0.51
Group: PE	−2.833e-02	7.394e-02	−0.38
Group: AE	7.632e-02	7.709e-02	0.99
TrialNr * congruent	−3.757e-04	1.028e-04	−3.65
TrialNr * incongruent	−4.274e-06	1.029e-04	−0.04
TrialNr * GE	6.245e-04	1.195e-04	5.23*
TrialNr * AE	6.839e-04	1.246e-04	5.49*
TrialNr * PE	5.071e-04	1.156e-04	4.39*
Congruent * GE	−1.470e-02	5.944e-02	−0.25
Incongruent * GE	−6.239e-02	5.073e-02	−1.23
Congruent * PE	4.655e-02	6.601e-02	0.71
Incongruent * PE	−5.133e-02	5.655e-02	−0.91
Congruent * AE	9.329e-02	5.612e-02	1.66
Incongruent * AE	−1.013e-01	4.777e-02	−2.12*
TrialNr * congruent * GE	4.552e-05	2.919e-04	0.16
TrialNr * incongruent * GE	−4.563e-04	2.923e-04	−1.56
TrialNr * congruent * PE	7.264e-05	2.822e-04	0.26
TrialNr * incongruent * PE	−3.725e-04	2.827e-04	−1.32
TrialNr * congruent * AE	1.972e-04	3.043e-04	0.65
TrialNr * incongruent * AE	−1.752e-04	3.037e-04	−0.58

*Note that the reference levels for this model are the control condition (for congruency) and the monolingual group (for group). Thus, the fixed effect of “congruency: congruent” compares control vs. congruent (i.e., facilitation), the fixed effect of “group: AE” compares monolinguals vs. AE, etc. Significant fixed effects (t > 2) are marked with an asterisk*.

Group comparisons (Table 6) revealed significantly smaller interference effects for AE bilinguals (*M* = 34 ms, *SE* = 6 ms) compared to monolinguals (*M* = 74 ms, *SE* = 8 ms; *z* = 2.12; *d* = 1.34; Figure 2A). There were no differences between the groups when collapsing over all congruencies (Figure 1E). However, as in the L1, to further examine non-conflict-specific effects of executive processing between groups, we also compared RTs of the control conditions between groups for the English Stroop task. As in the L1 data, a separate mixed effects model was fit using only trial number and group as fixed effects. Random effects included random intercepts for subject and item and random slopes for the effect of group by item, as well as interactions between trial number and group. The results did not show any differences between groups on the control RTs in the L2 Stroop task (all *z*'s < 2; Figure 1F).

**Table 6:** **Fixed effects of group comparisons for L2 Stroop RTs (based on model presented in Table 5)**.

	**Estimate**	***SE***	***z*-value**
**GLOBAL RTs**			
Monolingual vs. German	−3.844e-02	7.548e-02	−0.509
Monolingual vs. Polish	−2.833e-02	7.394e-02	−0.383
Monolingual vs. Arabic	7.632e-02	7.709e-02	0.990
German vs. Polish	−1.011e-02	7.028e-02	−0.144
German vs. Arabic	1.148e-01	7.598e-02	1.510
Polish vs. Arabic	1.047e-01	7.382e-02	1.418
**INTERFERENCE**			
Monolingual vs. German	−6.239e-02	5.073e-02	−1.230
Monolingual vs. Polish	−5.133e-02	5.655e-02	−0.908
Monolingual vs. Arabic	−1.013e-01	4.777e-02	−2.122*
German vs. Polish	−1.106e-02	3.331e-02	−0.332
German vs. Arabic	−3.896e-02	4.613e-02	−0.845
Polish vs. Arabic	−5.001e-02	4.744e-02	−1.054
**FACILITATION**			
Monolingual vs. German	−1.470e-02	5.944e-02	−0.247
Monolingual vs. Polish	4.665e-02	6.601e-02	0.707
Monolingual vs. Arabic	9.329e-02	5.612e-02	1.662
German vs. Polish	−6.134e-02	3.960e-02	−1.549
German vs. Arabic	1.080e-01	5.423e-02	1.991
Polish vs. Arabic	4.664e-02	5.564e-02	0.838

*Note that the monolingual comparisons are identical to those presented in Table 5. Significant effects (z > 2) are marked with an asterisk*.

### Simon task

**Figure 3:**
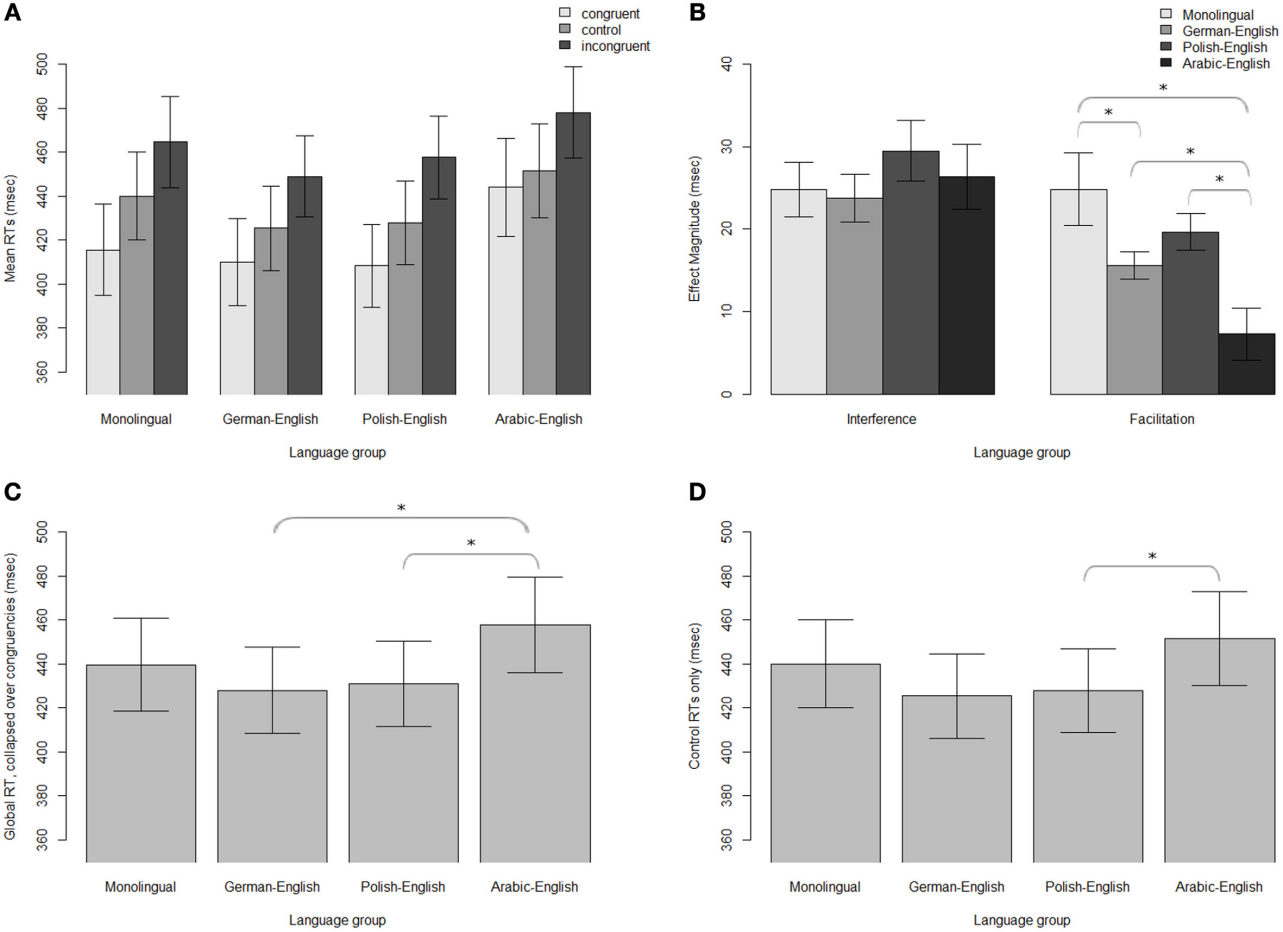
**Simon task data**. **(A)** Mean RTs for each group and congruency; **(B)** Interference and facilitation effects; **(C)** Mean RTs, collapsed over all congruencies; **(D)** Mean RTs for the control condition only. Significant differences between groups (*z* > 2) are indicated with an asterisk.

The results from the Simon task are presented in Figure 3. The final model for the RTs is presented in Table 7. The results revealed a significant interference effect when collapsed over groups, such that RTs were slower for incongruent trials (*M* = 462 ms, *SE* = 10 ms) than control trials (*M* = 435 ms, *SE* = 10 ms; *t* = 3.38; Table 7, Figure 3A). There was also a significant facilitation effect, with significantly faster RTs for congruent trials (*M* = 419 ms, *SE* = 10 ms) than control trials (*t* = 2.57).

Table 7**Results of the mixed-effects analysis of the Simon RTs for bilinguals and monolinguals**.

**Table:** 

**Random effects**		**Variance**	***SD***
Subject	Intercept	0.046181	0.21490
	congruent	0.001691	0.04112
	incongruent	0.002902	0.05387
Item	Intercept	0.001705	0.04129
Residual		0.145339	0.38123

**Table:** 

**Fixed effects**	**Estimate**	***SE***	***t*-value**
Intercept	−2.362+00	3.007e-02	−78.56*
TrialNr	−6.352e-04	2.504e-05	−25.37*
Congruency: congruent	−1.079e-01	4.201e-02	−2.57*
Congruency: incongruent	1.427e-01	4.222e-02	3.38*
Group: GE	−4.325e-02	7.111e-02	−0.61
Group: PE	−4.830e-02	3.872e-02	−0.70
Group: AE	1.095e-01	7.311e-02	1.50
TrialNr * congruent	4.303e-05	5.936e-05	0.72
TrialNr * incongruent	3.466e-05	6.029e-05	0.57
TrialNr * GE	−3.080e-04	7.524e-05	−4.09*
TrialNr * PE	−4.657e-05	7.349e-05	−0.63
TrialNr * AE	2.917e-05	7.676e-05	0.38
Congruent * GE	5.201e-02	2.257e-02	2.30*
Incongruent * GE	8.025e-03	2.556e-02	0.31
Congruent * PE	3.619e-02	2.194e-02	1.65
Incongruent * PE	3.828e-02	2.486e-02	1.54
Congruent * AE	1.078e-01	2.315e-02	4.66*
Incongruent * AE	5.219e-03	2.624e-02	0.20
TrialNr * congruent * GE	1.037e-04	1.751e-04	0.59
TrialNr * incongruent * GE	−2.221e-04	1.786e-04	−1.24
TrialNr * congruent * PE	8.052e-05	1.703e-04	0.47
TrialNr * incongruent * PE	−2.951e-04	1.743e-04	−1.69
TrialNr * congruent * AE	−2.368e-04	1.792e-04	−1.32
TrialNr * incongruent * AE	−1.627e-04	1.830e-04	−0.89

*Note that the reference levels for this model are the control condition (for congruency) and the monolingual group (for group). Thus, the fixed effect of “congruency: congruent” compares control vs. congruent (i.e., facilitation), the fixed effect of “group: AE” compares monolinguals vs. AE, etc. Significant fixed effects (t > 2) are marked with an asterisk*.

Group comparisons (Table 8) showed significantly greater facilitation effects for monolinguals (*M* = 25 ms, *SE* = 4 ms) compared to both AE bilinguals (*M* = 7 ms, *SE* = 3 ms; *z* = 4.66; *d* = 1.22) and GE bilinguals (*M* = 16 ms, *SE* = 2 ms; *z* = 2.30; *d* = 0.66; Figure 3B). AE bilinguals showed smaller facilitation effects than GE (*z* = 2.54; *d* = 0.85) and PE (*M* = 20 ms, *SE* = 2 ms; *z* = 3.36; *d* = 1.23) bilinguals.

**Table 8:** **Fixed effects of group comparisons for Simon RTs (based on model presented in Table 7)**.

	**Estimate**	***SE***	***z*-value**
**GLOBAL RTs**			
Monolingual vs. German	−4.325e-02	7.111e-02	−0.608
Monolingual vs. Polish	−4.830e-02	6.872e-02	−0.703
Monolingual vs. Arabic	1.095e-01	7.311e-02	1.497
German vs. Polish	5.051e-03	6.763e-02	0.075
German vs. Arabic	1.527e-01	7.209e-02	2.119*
Polish vs. Arabic	1.578e-01	6.973e-02	2.262*
**INTERFERENCE**			
Monolingual vs. German	8.025e-03	2.556e-02	0.314
Monolingual vs. Polish	3.828e-02	2.486e-02	1.540
Monolingual vs. Arabic	5.219e-03	2.624e-02	0.199
German vs. Polish	−3.026e-02	2.346e-02	−1.290
German vs. Arabic	−2.806e-03	2.493e-02	−0.113
Polish vs. Arabic	−3.306e-02	2.421e-02	−1.366
**FACILITATION**			
Monolingual vs. German	5.201e-02	2.257e-02	2.304*
Monolingual vs. Polish	3.619e-02	2.194e-02	1.650
Monolingual vs. Arabic	1.078e-01	2.315e-02	4.656*
German vs. Polish	1.582e-02	2.067e-02	0.765
German vs. Arabic	5.579e-02	2.196e-02	2.540*
Polish vs. Arabic	7.161e-02	2.131e-02	3.361*

*Note that the monolingual comparisons are identical to those presented in Table 7. Significant effects (z > 2) are marked with an asterisk*.

There was also a global RT effect such that, when comparing between groups when collapsing over all congruencies, AE bilinguals (*M* = 458 ms, *SE* = 22 ms) were significantly slower than GE bilinguals (*M* = 428 ms, *SE* = 20 ms; *z* = 2.12; *d* = 0.34) and PE bilinguals (*M* = 431 ms, *SE* = 19 ms; *z* = 2.26; *d* = 0.30; Figure 3C). To further examine non-conflict-specific effects of executive processing between groups, we also compared RTs of the control conditions between groups for the Simon task. For this analysis, a separate mixed effects model was fit using only trial number and group as fixed effects. Random effects included random intercepts for subject and item, as well as interactions between trial number and group. The results showed significantly longer control RTs for AE bilinguals (*M* = 452 ms, *SE* = 21 ms) compared to PE bilinguals (*M* = 428 ms, *SE* = 19 ms; *z* = 2.11; *d* = 0.27; Figure 3D).

### Reliability estimates

We also performed reliability estimates for all of the measures discussed here (results are presented in the Supplementary Tables S1, S2). Specifically, we calculated the mean RTs for odd and even trials for each subject, then correlated these means over each group (using Spearman correlations) and corrected them using the Spearman-Brown prophecy formula: (2^*^rho)/(1+rho). These estimates were calculated for the global RTs and the control RTs for each group and language (Supplementary Table S1). We also calculated reliability estimates for the interference and facilitation effects, using a calculation for reliability of a difference score (see Chiou and Spreng, 1996; Hughes et al., 2014; Supplementary Table S2). All of these values fell at or above 0.9, indicating high reliability of our Stroop and Simon measures.

## Discussion

The current study investigated the relationship between script similarity and bilingual executive control. Two hypotheses were proposed. Hypothesisx 1, based on the BIA+ model, predicted a positive relationship, with more effective executive control abilities associated with increasing language similarity such that same-script bilinguals would show the largest bilingual advantages. In contrast, Hypothesis 2, based on the results of previous literature, predicted a negative relationship, with more effective executive control associated with decreasing language similarity such that different-script bilinguals would show the largest bilingual advantages. To evaluate these hypotheses, three groups of bilinguals whose native languages had varying similarity with English (German, Polish, and Arabic), as well as a group of monolinguals, performed a Stroop task and a non-linguistic Simon task. The groups were compared on the magnitude of interference and global RT effects to evaluate the BICA and BEPA hypotheses, respectively.

### Interference and global RT effects

In the Stroop task, in both the L1 and L2 monolinguals showed numerically larger interference effects compared to the bilingual groups. However, there was only a significant bilingual interference advantage when comparing monolinguals to the Arabic-English bilinguals in the L1 and L2. Between bilinguals, Arabic bilinguals showed significantly smaller interference effects than Polish bilinguals in the L1; there were no differences between bilingual groups in the L2. However, there were no group differences in Simon interference effects, contradictory to the predictions of the BICA and to previous literature that has observed a bilingual advantage on this task (Bialystok et al., 2004, 2005b; Bialystok, 2006; Bialystok et al., 2008; Martin-Rhee and Bialystok, 2008).

Global RT effects were evaluated by both collapsing over all congruencies and by comparing only the control condition between groups. Across both the Stroop and Simon tasks, a similar pattern occurred in both types of comparisons such that numerically, Arabic-English bilinguals showed the longest RTs of all four groups. Statistically, Arabic-English bilinguals had significantly longer RTs compared to Polish-English bilinguals in the L1 Stroop task, and compared to both the Polish and German bilinguals in the Simon task.

Taken together, this data thus revealed incompatible results. Overall, Arabic-English bilinguals showed the smallest Stroop interference effects in both L1 and L2, which is in line with the predictions of Hypothesis 2, yet there were no group differences in Simon interference effects. In contrast, longer global RTs in both the Stroop and Simon tasks were observed for Arabic-English bilinguals, which is in line with the predictions of Hypothesis 1. These contradictory results can be explained by considering that the Stroop interference effects were likely driven by script similarity. In a Stroop task, which is linguistically-based, the input of the color word will lead to cross-linguistic activation of the word in the alternative language. However, because the two words are translation equivalents, or non-identical cognates (see Dijkstra et al., 2010), and are linked to the same semantic concept, they will elicit a facilitation effect (similar to the cognate facilitation effect) leading to faster and stronger activation of the concept of the word(s). In same-script bilinguals, the stronger activation of the concept (arising from greater cross-linguistic facilitation) will in turn lead to stronger conflict with the incongruent concept (arising from the color stimulus), generating larger Stroop interference effects. In contrast, different-script bilinguals will have less cross-linguistic facilitation, leading to a relatively weaker word concept to interfere with the color concept, and consequently smaller interference effects. Note that this also predicts larger facilitation effects for same-script bilinguals compared to different-script bilinguals, as are observed in the current data. This is a tentative interpretation, as there are currently no models of the bilingual Stroop task using a manual response; although these patterns replicate previous findings with the bilingual Stroop task (Brauer, 1998; van Heuven et al., 2011), these studies used a vocal response which also introduces the issue of production.

Although consistent with previous data, these cross-linguistic influences in Stroop interference effects also confound interpretations of executive control differences between bilingual groups. For this reason, the Simon task was included as a non-linguistic assessment of executive control. Because no differences in Simon interference effects occurred between groups, we cannot conclude the presence of an inhibitory control advantage in any group. However, Arabic-English bilinguals had longer global RTs compared to German-English and Polish-English bilinguals. This suggests less effective executive processing abilities for different-script bilinguals compared to similar-script bilinguals, as (partially) predicted by Hypothesis 1. Importantly, these patterns of global RT were consistent across both tasks, and similar patterns occurred when collapsing across congruencies and when comparing the control conditions alone (although effects were only statistically significant when including all congruencies). The fact that these consistent patterns also occurred in the absence of linguistic stimuli (in the Simon task) or conflicting/facilitating information (in the control condition of both tasks) suggests that these effects were not driven by explicit orthographic influences, but arose as a secondary result of the amount of cross-linguistic overlap for each bilingual group. Given these consistent patterns, we conclude that same-script bilinguals experience more effective cognitive control compared to different-script bilinguals, due to the relatively greater amount of cross-linguistic activation from their two languages.

There could be a number of alternative explanations for our finding that Arabic-English bilinguals have slower overall RTs than the other three groups. For instance, these individuals may have a lower broad speed of processing compared to the other groups tested; as we did not include a baseline measure of processing speed, such as an entire block of non-conflict control trials, we cannot completely rule this out. Furthermore, additional cultural or individual variables such as age (the Arabic bilinguals were slightly older than the other groups), less experience with university research labs, a different strategy of performing the task, etc., could also account for these differences. We note that the use of mixed effects modeling, and specifically the inclusion of subject as a random effect, can take subject variability into account, although it cannot provide insight into the underlying causes of such variability. We therefore urge caution in the interpretation of these global RT differences, as there may be a number of reasons why Arabic-English bilinguals were slower than the other groups which should be investigated in future studies (see further discussion in Section Limitations and Additional Considerations). However, in the context of the current study, we interpret the finding of slower RTs for the Arabic-English group as suggestive of less-effective executive control for different-script bilinguals compared to same-script bilinguals.

The finding of longer global RTs for different-script bilinguals contradicts the findings of Bialystok et al. (2005a) and Linck et al. (2008), who reported more effective executive control for different-script bilinguals compared to similar-script bilinguals. One contributor to these disparities may be differences in writing systems between the bilingual groups. Bialystok et al. (2005a) compared Cantonese-English bilinguals (logographic and alphabetic writing systems, respectively) to French-English bilinguals (both alphabetic writing systems). Similarly, Linck et al. (2008) contrasted Japanese-English bilinguals (Japanese has two scripts, the logographic kanji and the syllabic kana) with Spanish-English bilinguals (both alphabetic writing systems). In contrast, all of the languages tested here used alphabetic writing systems (although Arabic uses a different alphabet than German and Polish). It is possible that logographic writing systems have distinctive effects on executive control. For example, note that in the current data, there were no differences in interference or global RT effects between German and Polish bilinguals, despite the larger overlap in German and English than in Polish and English. This could indicate that writing system, rather than script, is the more influential factor in cross-linguistic effects: as German, Polish and English all use a similar alphabet, German-English and Polish-English bilinguals may experience similar cross-linguistic effects. Additional linguistic factors, such as the visuospatial properties of the scripts (e.g., the fact that Arabic is read right-to-left) and orthographic depth, may have also contributed to the observed patterns of results (Bar-Kochva, 2011; Taha et al., 2012). Given the limited literature investigating the influence of script overlap on the bilingual advantage, further research on these variables, especially the role of writing system, is warranted.

We also note that the current results do not support previous accounts of a bilingual advantage, as there were not consistently smaller interference or global RT effects for bilinguals compared to monolinguals (see also Section Limitations and Additional Considerations). To some degree, this supports previous reports showing that the bilingual advantage is highly elusive and sensitive to a number of individual and task-dependent factors (e.g., Hilchey and Klein, 2011; Paap and Greenberg, 2013; Duñabeitia et al., 2014; see Section Limitations and Additional Considerations). However, we emphasize that regardless of the presence of a bilingual advantage, the current results suggest that script similarity may modulate executive control abilities across bilingual groups. In fact, this variable may underlie some of the inconsistency in previous investigations of the bilingual advantage. Therefore, language similarity is an important variable that future studies of bilingual executive control need to take into account.

### Role of proficiency on executive control

As we have discussed above, the patterns of Stroop interference and facilitation effects in bilinguals may be due to the amount of orthographic overlap between bilingual language pairs, with greater cross-linguistic activation in same-script bilinguals leading to greater interference effects. However, an additional possibility might be that language proficiency is driving these effects. Reduced proficiency may lead to less “automatic” activation of a language, which would consequently result in reduced interference in color naming during the Stroop task. That is, the relatively smaller interference effects for Arabic-English bilinguals may be due to reduced cross-linguistic activation, or to reduced proficiency in this group. To address the possibility that L2 self-rated proficiency within bilingual groups affected the observed patterns of Stroop interference and facilitation effects in the L2, we incorporated language proficiency variables into the mixed model previously reported. Specifically, we used the structure of the final model from the Stroop L2 analyses (i.e., that of the model presented in Tables 5, 6) and ran it with only the bilingual subjects (because monolinguals do not have scores for L2 proficiency, this would have yielded NAs in the dataset, so they would not have been included in the modeling procedure). Using a backwards iteration procedure, we tested whether the inclusion of L2 proficiency (averaged over speaking, listening, reading, and writing) and L2 age of acquisition (AoA), and interactions of these variables with all fixed effects, significantly contributed to the model. The final model showed a significant contribution, and included L2 proficiency and AoA as fixed effects, and interactions of these variables with congruency and group. The results showed a significant difference in facilitation effects between Arabic and German bilinguals (*t* = 3.16; in the previous model without proficiency variables, this was a trend, *z* = 1.99). There was also an interaction between L2 proficiency and the difference in facilitation effects between Arabic and German bilinguals (*t* = 2.28), such that the magnitude of the facilitation effect increased with increasing L2 proficiency. This suggests that L2 proficiency does affect Stroop facilitation in the L2. Specifically, the smaller L2 facilitation effects for Arabic bilinguals compared to German bilinguals may have been due to the comparatively lower English proficiency for the former group.

We also ran a separate model including Y-lex adjusted scores (a measure of English language proficiency and vocabulary size) in the model used to compare monolingual and bilingual English Stroop performance (i.e., the model discussed in Tables 5, 6). Y-lex adjusted score was included as a fixed effect with interactions between all other fixed effects (congruency, group, and trial number). There was a significant effect of Y-lex score on overall facilitation effects (*t* = 2.12) such that facilitation effects increased with increasing proficiency, but no three-way interactions of this variable with group and congruency, which would have indicated that this variable significantly modulates interference and/or facilitation effects. Therefore, it seems that English vocabulary score does not strongly contribute to the observed patterns of Stroop performance in English between monolinguals and bilinguals.

Therefore, these proficiency analyses suggest that L2 proficiency may modulate Stroop L2 facilitation effects among the bilingual groups, although not when comparing monolinguals to bilinguals' L2. We caution that these interpretations are speculative, as we did not fully assess language proficiency in the current study. However, these results are intriguing, and future studies should systematically investigate the role of proficiency and script similarity in Stroop interference and facilitation effects.

### Limitations and additional considerations

The Stroop paradigm employed in this study was slightly atypical, as the color and word were spatially separated in order to enable the temporal separation for the SOA manipulation. There is some evidence that non-integration of the stimuli results in slightly decreased, but still, significant, conflict effects (e.g., MacLeod, 1991); therefore this manipulation could have influenced the observed patterns of results. As we mention above, it is possible that there are other contributing factors, besides script similarity, as to why the Arabic group had overall longer processing speeds than the other groups. Such individual variability may be one of the major problems faced in investigations of bilingual executive control, and could contribute to the increasingly common disparities in findings.

One major limitation is that the current results are based on a relatively small sample size in each group. Although our sample size is similar to those of previous studies on the bilingual advantage (e.g., Bialystok et al., 2004, 2005a; Carlson and Meltzoff, 2008; Emmorey et al., 2008), the issue of small sample size in the field of bilingualism is becoming more closely scrutinized (e.g., Hilchey and Klein, 2011; Paap and Greenberg, 2013). Indeed, *post-hoc* power analyses illustrated that we were underpowered to detect differences with small effect sizes, such as group differences in Simon interference. Such a limitation may explain the disparate results among previous investigations of the bilingual advantage. We therefore urge caution in interpreting our null findings, such as those in the Simon task. As this is one of the few studies to systematically investigate script similarity in bilingual executive control, we emphasize that our results are preliminary and need to be replicated and extended in future studies with larger sample sizes. Nevertheless, the take-home message from this work is that script similarity may modulate executive control abilities in bilinguals, and should be considered more thoroughly in future bilingualism research.

An additional consideration for future research is that the current study focused only the effects of orthographic overlap between bilinguals' language pairs, which can be explained by the BIA+ model. However, the BIA+ model is a model of word recognition and therefore accounts only for the written modality. As far as we are aware, current models of bilingual language control during production [e.g., the Inhibitory Control model (Green, 1998); the adaptive control hypothesis (Green and Abutalebi, 2013)] do not explicitly discuss how the phonological similarity between a bilingual's two languages might influence the needs for language control, although this could be implemented fairly easily. Cross-linguistic effects of phonology in word production have been documented in different-script bilinguals (Hoshino and Kroll, 2008; Wu and Thierry, 2010), but greater phonological overlap between languages could also lead to greater cross-linguistic activation in the word selection process. In the current study, German-English bilinguals may have experienced additional interference from cross-linguistic phonological information, since German and English share more phonological similarity than Polish and English or Arabic and English. In support of this proposal, additional data from a picture-naming task performed with the same bilinguals tested here has shown that German-English bilinguals experienced more cross-linguistic interference from phonology compared to the Polish-English bilinguals (Coderre and van Heuven, under review). Further work including a more systematic manipulation of phonological overlap between bilinguals' languages is needed to corroborate this proposal. Nevertheless, it may be the case that varying degrees of phonological overlap also contributes to executive control abilities in bilinguals and could modulate the bilingual advantage effect.

### Conclusions

In summary, the current data suggests that script similarity may affect bilingual cognitive control abilities, with bilinguals of similar languages experiencing more effective executive control compared to different-script bilinguals. As we did not find consistent evidence for a bilingual advantage as compared to monolinguals, with the advantage occurring primarily in the global RT effects, this suggests that script similarity may affect executive processing abilities more generally. Although preliminary, this work suggests that script similarity is another important aspect of individual variation to consider in past and future research into bilingual executive control.

### Conflict of interest statement

The authors declare that the research was conducted in the absence of any commercial or financial relationships that could be construed as a potential conflict of interest.
