# Supplementary material for: The effect of script similarity on executive control in bilinguals
Source: Front Psychol. 2014 Sep 29;5:1070. doi: 10.3389/fpsyg.2014.01070 (PMC4212224; doi:10.3389/fpsyg.2014.01070)
Supplement: Supplementary file 1 [file DataSheet1.DOCX]

Table S1: Spearman-Brown reliability correlations for control RTs (control condition only) and global RTs (collapsed over all congruencies), for each group, language, and task.

| **Group** | **Task** | **Spearman-Brown reliability correlation** | |
| --- | --- | --- | --- |
|  |  | **control RTs** | **global RTs** |
| German-English | Stroop L1 (German) | 0.93 | 0.98 |
|  | Stroop L2 (English) | 0.97 | 0.99 |
|  | Simon | 0.96 | 0.98 |
| Polish-English | Stroop L1 (Polish) | 0.96 | 0.95 |
|  | Stroop L2 (English) | 0.97 | 0.99 |
|  | Simon | 0.97 | 0.99 |
| Arabic-English | Stroop L1 (Arabic) | 0.95 | 0.99 |
|  | Stroop L2 (English) | 0.94 | 0.98 |
|  | Simon | 0.95 | 0.99 |
| Monolinguals | Stroop (English) | 0.97 | 0.99 |
|  | Simon task | 0.94 | 0.98 |

Table S2: Reliability of the difference scores^^[[1]](#footnote-1)^^ for interference and facilitation effects, for each group, language, and task.

| **Group** | **Language** | **Reliability of difference score** | |
| --- | --- | --- | --- |
|  |  | **Interference Effect** | **Facilitation Effect** |
| German-English | Stroop L1 (German) | 0.94 | 0.93 |
|  | Stroop L2 (English) | 0.95 | 0.96 |
|  | Simon | 0.96 | 0.96 |
| Polish-English | Stroop L1 (Polish) | 0.89 | 0.95 |
|  | Stroop L2 (English) | 0.94 | 0.95 |
|  | Simon | 0.97 | 0.95 |
| Arabic-English | Stroop L1 (Arabic) | 0.91 | 0.94 |
|  | Stroop L2 (English) | 0.94 | 0.94 |
|  | Simon | 0.96 | 0.95 |
| Monolinguals | Stroop (English) | 0.94 | 0.96 |
|  | Simon task | 0.94 | 0.94 |
|  |  |  |  |

1. Reliability estimates for difference scores were calculated based on the equation given in Chiou & Spreng (1996), and Hughes, Linck, Bowles, Koeth, & Bunting (2014):

   ρ_dd'_ = σ_x_^2^ ρ_xx'_ + σ_y_^2^ ρ_yy'_  - 2 ρ_xy_ σ_x_ σ_y_

   σ_x_^2^ + σ_y_^2^ - 2 ρ_xy_ σ_x_ σ_y_

   Where:

   ρ_dd'_  = Reliability of difference score

   ρ_xx’_ = Reliability of condition 1 (e.g. incongruent RTs)

   ρ_yy'_ = Reliability of condition 2 (e.g. control RTs)

   ρ_xy_ = Correlation between condition 1 and condition 2

   σ_x_^2^ = Variance of condition 1

   σ_y_^2^ = Variance of condition 2

   NB: Chiou & Spreng (1996) also provide an alternative formula if the variability and reliability of condition 1 and condition 2 are not equal, but this formula gives the same results as shown here. [↑](#footnote-ref-1)
